# The multifaceted roles of E3 ubiquitin ligases in osteoarthritis

**DOI:** 10.3389/fcell.2025.1665313

**Published:** 2025-08-22

**Authors:** Zheng Jin, Zhenhua Zhu, Jun Chen, Xiaopeng Jing, Jie Tan, Ji Zeng

**Affiliations:** ^1^ Department of Clinical Laboratory, Wuhan Fourth Hospital, Wuhan, China; ^2^ Department of Joint Surgery, Wuhan Fourth Hospital, Wuhan, China; ^3^ Orthopedic Department, Wuhan Fourth Hospital, Wuhan, China; ^4^ Orthopedic Laboratory, Orthopedic Department & Hubei Sports Medicine Center, Wuhan Fourth Hospital, Wuhan, China

**Keywords:** osteoarthritis, E3 ubiquitin ligases, ubiquitination, mechanisms, treatments

## Abstract

Osteoarthritis (OA) is the most widespread joint disorder worldwide. It is a major cause of lower limb mobility issues in the elderly. With the ongoing aging of the global population and the increasing prevalence of obesity, the disease burden associated with OA is expected to rise significantly. E3 ubiquitin ligases (E3s) play a crucial role in protein ubiquitination. They identify specific substrates and attach ubiquitin molecules to substrates, thus modulating protein stability, function, and cellular localization. E3s can be classified into three main types: RING-, HECT-, and RBR-type E3s. Growing evidence indicates that E3s affect OA by regulating the degradation of extracellular matrix (ECM) proteins and inflammatory responses. This review highlights the functions and underlying mechanisms of E3s in OA, aiming to provide new therapeutic insights for the treatment of OA.

## 1 Introduction

Osteoarthritis (OA) affects millions of people worldwide. It is a degenerative joint disease characterized by the breakdown of cartilage and underlying bone in the joints, leading to pain, stiffness, and reduced mobility (Arden and Nevitt, 2006). OA affects the hands, hips, knees, feet, and spine. The precise etiology of OA remains incompletely understood, but it is believed to result from a combination of age, gender, obesity, joint injury, genetics (He et al., 2020). With the aging of the population, the prevalence of OA is expected to increase significantly. Globally, 595 million people had OA in 2020, equal to 7·6% of the global population, and an increase of 132·2% in total cases since 1990 (GBD, 2021 Osteoarthritis Collaborators, 2023).

Ubiquitination is an important post-translational modification process. It involves a series of special enzymes that classify proteins within the cell, select target protein molecules, and then specifically modify these target proteins. Ubiquitin, a low-molecular-weight protein composed of 76 amino acids, is highly conserved and widely expressed in eukaryotic organisms (Agrata and Komander, 2025). Ubiquitination plays a key role in a variety of physiological processes (Husnjak and Dikic, 2012). Dysregulation of ubiquitination is closely related to many diseases, including cancer, neurodegenerative diseases, muscular dystrophy, immune disorders, and metabolic syndromes (Schwartz and Ciechanover, 2009). Modulation of the ubiquitination pathway is considered a promising therapeutic strategy for tumors (Wirth et al., 2020) and neurodegenerative diseases (Schmidt et al., 2021). The ubiquitination process involves a cascade of enzymes: ubiquitin-activating enzymes (E1s), ubiquitin-conjugating enzymes (E2s), ubiquitin ligases (E3s). E1s activate ubiquitin using ATP energy, the activated ubiquitin is transferred to E2s, E2s bind to E3s and E3s transfer ubiquitin to the target protein (Zheng and Shabek, 2017).

As described, E3s are key enzymes for ubiquitination modification, responsible for recognizing specific substrates and transferring ubiquitin onto the substrates (Foot et al., 2017). Currently, over 600 E3s have been identified. Based on structural characteristics, E3s are divided into three categories: the RING, HECT (homologous with E6-associated protein C-terminus), and RBR (RING between RING) family (Uchida and Kitagawa, 2016). RING family proteins have only one RING domain, which mainly serves as a bridge to connect E2s and substrate proteins, promoting ubiquitin transport (Lou et al., 2023). HECT family proteins have a common C-terminal HECT domain that mediates the transfer of ubiquitin from E2 to the target protein (Takeda et al., 2023). RBR family proteins have two RING domains, which are connected through the BR domain. Through these two RING domains, it interacts with E2s and substrates to complete protein ubiquitination (Pao et al., 2018).

Numerous studies have demonstrated that E3s hold significant importance in areas including oncology and infectious diseases (Kumar et al., 2024; Pellman et al., 2024). Furthermore, an increasing number of studies have highlighted the involvement of E3s in the progression of OA (Qi et al., 2022; Lin X. et al., 2021; Chen et al., 2022). In this review, we summarized the diverse roles of E3s in OA, explored then mechanisms, and examined the potential of E3s as therapeutic targets for OA treatment.

## 2 The pathogenesis and risk factors of OA

In OA, cartilage degeneration is mainly triggered by chondrocyte hypertrophy, the upregulation of enzymes that degrade the extracellular matrix (ECM), and a reduction in the synthesis of vital ECM components. These changes create an imbalance that impairs ECM production, causes cartilage to thin, and induces fibrotic alterations. Collectively, these factors worsen joint function (Maldonado and Nam, 2013). Matrix metalloproteinases (MMPs) are zinc-dependent endopeptidases that degrade ECM components and are involved in tissue remodeling, inflammation, and tumor invasion (Mustafa et al., 2022). MMP13 and MMP14 are two members of the MMP family. MMP13 is a collagenase that primarily degrades type I, II, and III collagens, which are the main components of the ECM (Chan et al., 2017). The expression of MMP13 is regulated by various cytokines and growth factors, like tumor necrosis factor (TNF)-α and interleukin-1β (IL-1β) (Alvarez et al., 2024). MMP14 can degrade a variety of ECM components, including collagens, fibronectin, and laminin. Due to its membrane-bound nature, MMP14 can locally degrade ECM on the cell surface, providing pathways for cell migration and invasion (Itoh, 2015). The expression of MMP14 is also regulated by various cytokines and growth factors, such as transforming growth factor (TGF)-β and epidermal growth factor (EGF) (Krstic and Santibanez, 2014). MMP14 can directly activate MMP13 (Knäuper et al., 2002), the upregulation of MMP13 leads to the degradation and destruction of articular cartilage, leading to the degradation of articular cartilage (Mukherjee and Das, 2024).

When stimulated by inflammatory cytokines such as TNF-α and IL-1β, or by mechanical stress, the nuclear factor-κB (NF-κB) signaling pathway is activated. This activation leads to an increase in both pro-inflammatory factors and ECM-degrading enzymes (e,g, MMP13). The elevated levels of these inflammatory factors further stimulate the NF-κB pathway, establishing a positive feedback loop that intensifies the progression of OA (Choi et al., 2019).

The Wnt/β-catenin signaling pathway also plays a crucial role in OA. The nuclear translocation of β-catenin induces the expression of runt-related transcription factor 2 (Runx2) and matrix metalloproteinases (MMPs), thereby driving chondrocyte hypertrophy and degradation of the ECM (Zeng et al., 2025; Feng et al., 2024).

Several factors are associated with an increased risk of developing OA, including obesity, female gender, advancing age, knee injuries, and participation in high-impact sports (Zhang and Jordan, 2010). While aging and OA are distinct biological processes, they are closely correlated statistically (He et al., 2024).

## 3 E3 ubiquitin ligases in OA

### 3.1 The RING E3 ubiquitin ligases

**FIGURE 1:**
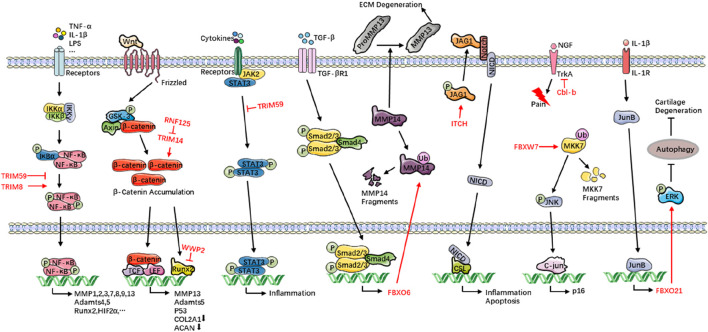
E3s orchestrate OA progression. Healthy chondrocytes continuously deposit COL2A1 and proteoglycans (e.g., aggrecan) to sustain the ECM and its biomechanical properties. In OA, chronic inflammatory cues and aberrant mechanical load skew chondrocyte metabolism toward catabolism: ECM synthesis is sharply curtailed while degradation surges. This I mbalance tips cartilage homeostasis toward net tissue loss. Collagenolytic MMP1, MMP3, MMP13 and proteoglycan-cleaving Adamts4 and Adamts5 are unleashed, eroding ECM integrity. Concomitant oxidative and ER stress, amplified by persistent inflammatory signaling, triggers chondrocyte apoptosis, depleting the resident cell pool and crippling intrinsic repair. E3s modulate these events with pathway-specific precision—directing NF-κB, Wnt/β-catenin, and autophagy circuits that govern chondrocyte survival, metabolic flux, and inflammatory output—thereby serving as pivotal regulators of OA onset and progression. TRIM, tripartite motif-containing; RNF125, ring finger protein 125; WWP2, WW domain-containing protein 2; FBXO, F-box only protein; ITCH, Itchy E3 ubiquitin protein ligase protein; FBXW7, F-box and WD repeat domain-containing 7; Cbl-b, Casitas B-lineage lymphoma b; OA, osteoarthritis; COL2A1, collagen type II alpha 1 chain; ECM, extracellular matrix; MMP, matrix metalloproteinase; Adamts, a disintegrin and metalloproteinase with thrombospondin motifs; TNF-α, tumor necrosis factor-α; IL-1β, interleukin-1β; LPS, lipopolysaccharide; IKK, IκB kinase; IκBα, inhibitor of κB alpha; NF-κB, nuclear factor kappa-B; Runx2, runt-related transcription factor 2; HIF2α, hypoxia-inducible factor 2 alpha; GSK-3, glycogen synthase kinase-3; Axin, axis inhibition protein; TCF, T-cell factor; LEF, lymphoid enhancer factor; ACAN, aggrecan; JAK2, Janus kinase 2; STAT, signal transducer and activator of transcription 3; TGF-β, transforming growth factor-β; JAG1, Jagged 1; NICD, Notch intracellular domain; CSL, CBF1/Su(H)/Lag-1; NGF, neurotrophic factors; TrkA, tropomyosin-related kinase A; MKK7, mitogen-activated protein kinase kinase 7; JNK, c-Jun N-terminal kinase; ERK, extracellular regulated protein kinase.

Casitas B-lineage lymphoma b (Cbl-b) is a RING-type E3 ubiquitin ligase (Fusco et al., 2025) that can ubiquitinate tropomyosin-related kinase A (TrkA), a key nerve growth factor receptor involved in pathological pain. Neurotrophic factors (NGF) enhance the excitability of sensory neurons by activating TrkA, thereby modulating pain perception (Mantyh et al., 2011). The ubiquitination of TrkA by Cbl-b exerts a continuous negative regulation on the protein level of TrkA and can alleviate OA pain (Chen et al., 2022) (Table 1; Figure 1).

**TABLE 1:** E3 ubiquitin ligases regulate OA.

Subgroup	Name	Target	Functions	References
RING	Cbl-b	TrkA	Alleviates OA pain through ubiquitination and degradation of TrkA.	Chen et al. (2022)
	HRD1	OS9	Might play a role in the proliferation of OA synovial cells.	Ye et al. (2018)
	TRIM59	ND	Inhibits of the NF-κB and JAK2/STAT3 signaling pathways.	Teng et al. (2020)
	TRIM8	ND	Knockdown of TRIM8 suppresses the NF-κB pathway and alleviates the inflammatory response in chondrocytes.	Liu et al. (2020)
	TRIM14	ND	Activates the Wnt/β-catenin and NF-κB pathways, thereby promoting inflammation.	Lv et al. (2023)
	RNF125	TRIM14	Promotes ubiquitination and degradation of TRIM14, inhibits β-catenin nuclear translocation, and blocks Wnt/β-catenin signaling pathway.	Lv et al. (2023)
HECT	WWP2	Runx2	Protects cartilage from OA by ubiquitinating and degrading Runx2, thereby inhibiting the induction of Adamts5.	Mok et al. (2019)
	SMURF1	ND	Downregulates the expression of Runx2, Sox9, and TGFBR1.	Schminke et al. (2021)
	SMURF2	ND	Downregulates the expression of Runx2 and TGFBR1.	Schminke et al. (2021)
		SIRT1	Binds to SIRT1 and promotes its ubiquitination and degradation, thereby reducing cartilage repair in OA.	Shi et al. (2021)
	ITCH	JAG1	Degrades of JAG1 thereby inhibits the Notch1 signaling pathway and alleviates articular cartilage damage.	Qi et al. (2022)
	HECTD1	Rubicon	Mediates Rubicon degradation and regulates chondrocyte autophagy, then reduces chondrocyte death.	Liao et al. (2023)
RBR	Parkin	ND	Reduces OXPHOS and increases glycolysis in bone metabolism.	Ma et al. (2024)
Others	FBXW7	MKK7	Mediates the degradation of MKK7, thereby alleviating OA through the regulation of the JNK signaling pathway.	Zhang et al. (2022)
	FBXO3	ND	Depletion of FBXO3 inhibits the expression of IL-18, IL-1β, and pyroptosis-related proteins.	Wang et al. (2022)
	FBXO6	MMP14	Reduces the expression of MMP-14 through ubiquitination and degradation, thereby inhibiting the proteolytic activation of MMP-13.	Wang et al. (2020)
	FBXO21	ERK	Interacts with and phosphorylates ERK to inhibit autophagy, alleviates anabolism, and enhances apoptosis and catabolism in rat chondrocytes.	Lin et al. (2021b)
	UFL1	ND	Promotes the survival of IL-1β-mediated chondrocytes, suppresses NF-κB signaling pathway.	Yang et al. (2020)

ND, not determined.

HMG-CoA reductase degradation protein 1 (HRD1) is an E3 ubiquitin ligase involved in the endoplasmic reticulum (ER) membrane. The cytoplasmic C-terminal region of HRD1 contains a RING finger domain, which is essential for its function as an E3 ubiquitin ligase (Miyamoto et al., 2019). It mainly functions in the ER-associated degradation (ERAD) pathway, where it identifies and ubiquitinates misfolded or unfolded proteins, thereby directing them to the proteasome for degradation (Doroudgar et al., 2015). As a substrate of HRD1, osteosarcoma amplified (OS) nine is significantly upregulated under endoplasmic reticulum (ER) stress conditions and acts as an essential component in the cellular response to ER stress. It helps restore normal ER function by recognizing and degrading misfolded proteins (Ward et al., 2018). HRD1 regulates the stability of OS9.1 and OS9.2. In OA and rheumatoid arthritis (RA) tissues, The expression of OS9 is negatively correlated with HRD1 (Ye et al., 2018). However, the specific mechanisms underlying this correlation remain to be further explored (Table 1).

Tripartite motif-containing (TRIM) 59 is classified as a member of the RING family of E3 ubiquitin ligases due to its RING domain (Jin et al., 2024). In OA cartilage, TRIM59 is downregulated. It regulates the NF-κB and Janus kinase 2 (JAK2)/signal transducer and activator of transcription 3 (STAT3) signaling pathways, resulting in an imbalance in ECM metabolism. Also, this promotes the production of proinflammatory cytokines, apoptosis, and decreased cell viability, while simultaneously increasing the synthesis of type II collagen (COL2A1) and aggrecan and inhibiting MMP13 (Teng et al., 2020) (Table 1; Figure 1).

In contrast to TRIM59, TRIM8, which also belongs to the TRIM protein family, is overexpressed in OA chondrocytes (Liu et al., 2020). TRIM8 knockdown reduces nitric oxide (NO), prostaglandin E_2_ (PGE_2_), inducible nitric oxide synthase (iNOS), cyclooxygenase-2 (COX-2), TNF-α, and IL-6 production, while rescuing the IL-1β-induced decline in aggrecan and COL2A1 expression in chondrocytes. Further investigation reveals that TRIM8 knockdown blocks IL-1β-mediated activation of the NF-κB signaling pathway in OA (Liu et al., 2020) (Table 1; Figure 1).

Ring finger protein (RNF) 125 is downregulated in the cartilage tissues of OA rats, its overexpression alleviated OA. Overexpression of RNF125 in chondrocytes inhibits IL-1β-induced degradation of the ECM, enhances cell viability, promotes the expression of COL2A1 and aggrecan (ACAN), and suppresses the expression of MMP1, MMP13, and a disintegrin and metalloproteinase with thrombospondin motifs 5 (Adamts5). Mechanistically, RNF125 facilitates the ubiquitination and degradation of the TRIM14 protein. TRIM14 exacerbates the progression of OA by activating the Wnt/β-catenin and NF-κB signaling pathways, thereby promoting inflammatory responses and degradation of the cartilage matrix. By targeting TRIM14, a member of the RING family, RNF125 prevents the nuclear translocation of β-catenin, and blocks the activation of the Wnt/β-catenin signaling pathway (Lv et al., 2023) (Table 1; Figure 1).

### 3.2 The HECT E3 ubiquitin ligases

WW domain-containing protein 2 (WWP2), containing approximately 870 amino acids, is composed of three main domains: the N-terminal C2 domain, four tandem WW domains, and the C-terminal HECT domain (Zhang Y. et al., 2020). It belongs to the neural precursor cell expressed developmentally downregulated 4 (NEDD4) subfamily and regulates the stability, activity, or subcellular localization of target proteins through ubiquitination (You et al., 2024). In articular cartilage, WWP2 is highly expressed. Mice develop OA when WWP2 is deficient, downregulated, or fails to function as an E3 ubiquitin ligase. Conversely, injecting WWP2 mRNA into mouse joints can mitigate the severity of experimentally induced OA. Mechanistic studies reveals that the loss of WWP2 E3 ligase activity results in upregulation of Runx2-Adamts5 signaling pathway in articular cartilage. WWP2 protects cartilage from OA by ubiquitinating and degrading Runx2, thereby inhibiting the induction of Adamts5 (Mok et al., 2019) (Table 1; Figure 1).

SMAD specific E3 ubiquitin protein ligase 1 (SMURF1) and SMURF2 share significant structural and functional similarities. Their domain architecture, including the C2, WW, and HECT domains, enables them to perform similar roles in ubiquitination and degradation of target proteins. Both proteins regulate critical signaling pathways, such as TGF-β/BMP and Wnt/β-catenin, and influence cellular processes (Kavsak et al., 2000; Fu et al., 2020). SMURF1 and SMURF2 are expressed in OA tissues. In chondrogenic progenitor cells (CPCs) and meniscus progenitor cells (MPC), SMURF1 reduces Runx2, Sox9, and TGFBR1, SMURF2 also reduces the levels of Runx2 and TGFBR1, while Sox9 remains unaffected. The identity and function of the target protein are still not well understood (Schminke et al., 2021). In another study, SMURF2 can mediate the ubiquitination and degradation of silent information regulator factor 2-related enzyme 1 (SIRT1) (Shi et al., 2021). In cartilage formation, SIRT1 interactes with Sox9 to regulate its acetylation, enhancing Sox9 transcriptional activity and collagen 2 (α1) transcription (Dvir-Ginzberg et al., 2008; Qu et al., 2016). Thus, SMURF2 degrades SIRT and reduces cartilage repair in OA (Table 1).

The Itchy E3 ubiquitin protein ligase protein (ITCH) is a HECT domain-containing E3 ubiquitin ligase and belongs to the NEDD4 family. ITCH can ubiquitinate and degrade FLICE inhibitory protein (FLIP, an inhibitor of caspase-8), thereby promoting apoptosis (Holloway et al., 2024). It can also ubiquitinate and degrade cyclin-dependent kinase 4 (CDK4) and ribose-5-phosphate isomerase 23A (RPI23A), leading to cell cycle arrest in the G1 phase and thus inhibiting tumor (GBD, 2021 Osteoarthritis Collaborators, 2023). In OA, ITCH binds to the Jagged 1 (JAG1) protein via the WW-PPXY motif and degrades JAG1, thereby inhibiting the Notch1 signaling pathway and alleviating articular cartilage damage (Qi et al., 2022) (Table 1; Figure 1).

The HECT domain E3 ubiquitin protein ligase 1 (HECTD1) contains N-terminal ankyrin repeats, a MIB domain, and a C-terminal HECT domain. The HECT domain determines the specificity of the target protein (Fang et al., 2018). Its main function is to mediate protein ubiquitination. Studies have shown that HECTD1 expression levels are elevated during the G2/M phase and S phase of the cell cycle, where it facilitates cell proliferation and contributes to cell cycle regulation (Vaughan et al., 2022; Salas et al., 2023). The downregulation of HECTD1 in OA cartilage samples has been observed, and it has been demonstrated that HECTD1 can alleviate OA. The degradation of Rubicon mediates by HECTD1 regulates chondrocyte autophagy, reduces chondrocyte death, and mitigates the progression of OA (Liao et al., 2023) (Table 1).

### 3.3 The RBR E3 ubiquitin ligases

Parkin is the principal pathogenic protein in Parkinson’s disease, and its encoding gene has emerged as a major genetic risk factor for neurodegenerative disorders (Klein and Westenberger, 2012). Parkin is an E3 ubiquitin ligase, comprising Ubl, RING0, RING1, IBR, and RING2 domains. The Ubl domain is crucial for its function (Cruts et al., 2012). As an RBR-type E3 ubiquitin ligase, Parkin regulates mitochondrial homeostasis and protein quality control through ubiquitination, and its dysfunction represents a core pathogenic mechanism in Parkinson’s disease (PD) (Seirafi et al., 2015). Loss of Parkin function leads to toxic protein accumulation, mitochondrial impairment, and dopaminergic neuron death, accounting for over 50% of early-onset PD cases (Filograna et al., 2024). Parkin improves OA associated with aging by reducing the energy metabolism shift from glycolysis to oxidative phosphorylation (OXPHOS) in bone metabolism and increasing glycolysis (Ma et al., 2024) (Table 1).

### 3.4 The other E3 ubiquitin ligases

F-box and WD repeat domain-containing 7 (FBXW7) is an F-box protein and serves as the substrate recognition component of the Skp1-Cullin1-F-box (SCF) complex (Zhang Z. et al., 2020). FBXW7 recognizes specific phosphorylated substrates through its WD40 domain and inhibits tumorigenesis by promoting the ubiquitination and degradation of these substrates (Chen et al., 2023). In the cartilage of OA patients, the expression of FBXW7 is reduced. The absence of FBXW7 in chondrocytes exacerbates the progression of OA. FBXW7 mediates the degradation of mitogen-activated protein kinase kinase 7 (MKK7), thereby alleviating OA through the regulation of the c-Jun N-terminal kinase (JNK) signaling pathway (Zhang et al., 2022) (Table 1; Figure 1).

F-box only protein 3 (FBXO3) contains an F-box domain, which enables it to function as a component of the SCF complex and participate in the ubiquitination process of proteins (Gao et al., 2022). In the cartilage of OA patients, the expression of FBXO3 is reduced. The absence of FBXO3 exacerbates the progression of OA, manifested by an increase in the expression of IL-18, IL-1β, and pyroptosis-related proteins. Upregulation of miR-219a-5p can inhibit the expression of FBXO3, thereby alleviating the pathological progression of OA (Wang et al., 2022) (Table 1).

FBXO6 and FBXO3 are both members of the F-box protein family and play important roles in OA. FBXO6 inhibits MMP14 through ubiquitination and degradation, thereby inhibiting the proteolytic activation of MMP13. Knockout of FBXO6 accelerates the progression of OA in mice. The TGF-β-SMAD2/3 signaling pathway can upregulate the transcription of FBXO6 (Wang et al., 2020) (Table 1; Figure 1).

Unlike FBXO3, the expression of FBXO21 is higher in the cartilage of patients with OA. Additionally, FBXO21 exerts its effects on cartilage degeneration by inhibiting autophagy through the phosphorylation of extracellular regulated protein kinases (ERK) in chondrocytes. Meanwhile, JunB directly targets the promoter of FBXO21 and upregulates its expression (Lin Z. et al., 2021) (Table 1; Figure 1).

Ubiquitin-fold modifier 1 (UFM1)-specific ligase 1 (UFL1) is an E3 ligase that participates in the UFM1-mediated ubiquitin-like modification process (UFMylation). UFM1 is a ubiquitin-like protein, and UFL1 is the sole E3 ligase in the UFM1 modification system. However, its structure does not show obvious homology with known RING or HECT-type E3 ligases (Witting and Mulder, 2021). In OA, the increased expression of UFL1 promotes the survival of IL-1β-mediated chondrocytes, while inhibiting the expression of NO, PGE_2_, iNOS, COX-2, Adamts4, Adamts5, MMP3, and MMP13, and suppressing the NF-κB signaling pathway (Yang et al., 2020) (Table 1).

## 4 Discussion and conclusions

This review systematically delineates the multifaceted regulatory roles of E3s in the initiation and progression of OA, highlighting their central position in governing chondrocyte metabolism, inflammatory responses, apoptosis, and ECM homeostasis. By integrating the functions of RING-, HECT-, RBR-, and other classes of E3s, we reveal that these enzymes are not only pivotal participants in OA pathogenesis but also promising therapeutic targets for intervention.

Although the pathogenesis of OA is complex—involving mechanical stress, inflammatory cytokines, and metabolic dysfunction—E3s exert “precision control” over these factors at the molecular level through the ubiquitin–proteasome system (UPS). E3s modulate OA by exerting anti-inflammatory and anti-catabolic effects: TRIM59, RNF125, and FBXO3 suppress NF-κB and Wnt/β-catenin signalling, thereby reducing expression of matrix-degrading enzymes such as MMPs and Adamts5 (Teng et al., 2020; Lv et al., 2023; Wang et al., 2022). Conversely, E3s promote anabolism and repair: WWP2 ubiquitinates and degrades Runx2, thereby repressing Adamts5 and sustaining synthesis of COL2A1 and aggrecan (Mok et al., 2019). Moreover, HECTD1-mediated degradation of Rubicon activates autophagy and delays chondrocyte apoptosis (Liao et al., 2023), whereas Cbl-b alleviates OA-associated neuropathic pain by ubiquitinating and degrading the nerve-growth-factor receptor TrkA (Chen et al., 2022). Together, these mechanisms form an integrated E3-regulatory network that maintains cartilage homeostasis and halts OA progression.

Multiple E3 ligases have already demonstrated therapeutic efficacy in animal models; for example, intra-articular injection of WWP2 mRNA significantly attenuates OA progression in mice (Mok et al., 2019). Owing to their intrinsic substrate specificity, E3s can, in principle, circumvent the broad off-target effects associated with conventional anti-inflammatory therapies. A single E3 may simultaneously modulate several OA-relevant pathways—such as NF-κB, Wnt/β-catenin, and autophagy—thereby offering a “one-target, multi-pathway” advantage (Lv et al., 2023; Mok et al., 2019). However, mRNA-based therapies also have many limitations. mRNA is inherently unstable and susceptible to degradation by nucleases, which results in a short half-life and consequently affects the therapeutic efficacy (Shi et al., 2024). Exogenous mRNA may be recognized as a foreign substance by the immune system, activating Toll-like receptors (TLRs) and cytoplasmic nucleic acid receptors, thereby triggering immune responses that accelerates the inactivation of mRNA (Shi et al., 2024). Although chemical modification can reduce immunogenicity, completely eliminating the immune response remains a challenge (Nelson et al., 2020; Muslimov et al., 2023).

Nevertheless, achieving efficient and stable expression of E3s within articular cartilage remains an unmet challenge. The safety and delivery efficiency of vectors such as adeno-associated virus (AAV), lipid-encapsulated mRNA, or nanoparticle platforms require further validation (Mok et al., 2019; Liao et al., 2023). Additionally, certain E3s can exert context-dependent roles—for instance, when Parkin function is lost, damaged mitochondria cannot be effectively cleared, leading to further mitochondrial dysfunction and excessive production of reactive oxygen species, ultimately cause injury to dopaminergic neurons (Klein and Westenberger, 2012). Chondrocytes exhibit a shift in energy metabolism from glycolysis to OXPHOS during the aging process, and Parkin can reverse this process, thereby improving aging-related OA (Ma et al., 2024). Rigorous delineation of tissue-specific regulatory mechanisms is therefore essential before clinical translation.

In future studies, single-cell RNA-seq and proteomics can be employed to generate comprehensive E3s expression atlases and functional networks across distinct OA subtypes. Rational design of small-molecule E3 activators or inhibitors, together with CRISPR-based gene-regulation systems, will accelerate the development of E3s-targeted drugs or gene therapies. Moreover, the intricate interplay between E3s and non-coding RNAs or epigenetic modifiers—exemplified by miR-219a-5p-mediated repression of FBXO3 (Wang et al., 2022)—indicates that E3s operate within a higher-order regulatory hierarchy that warrants deeper exploration. Validation of these therapeutic concepts should be carried out in large-animal OA models (e.g., porcine or canine) that more closely recapitulate human disease.

In summary, E3s function as versatile “molecular switches” that control protein homeostasis, signal transduction, and cell fate in OA. With continued advances in gene editing, nanoscale delivery, and precision medicine, E3-based strategies are poised to translate from mechanistic insights into clinically effective, highly targeted, and low-toxicity therapies for OA patients.
